# Predictors of fear of childbirth and depressive symptoms among pregnant women: a cross-sectional survey in Pwani region, Tanzania

**DOI:** 10.1186/s12884-021-04169-7

**Published:** 2021-10-19

**Authors:** Agnes Fredrick Massae, Margareta Larsson, Sebalda Leshabari, Columba Mbekenga, Andrea Barnabas Pembe, Agneta Skoog Svanberg

**Affiliations:** 1 Department of Women’s and Children’s Health, Uppsala University, Uppsala, Sweden; 2 Department of Community Health Nursing, Muhimbili University of Health and Allied Sciences, Dar es Salaam, Tanzania; 3 School of Nursing and Midwifery, The Aga Khan University, Dar es Salaam, Tanzania; 4 Department of Obstetrics and Gynaecology, Muhimbili University of Health and Allied Sciences, Dar es Salaam, Tanzania

**Keywords:** Fear of childbirth, Depressive symptoms, Pregnancy, Childbirth, Prevalence, Predictors, Tanzania

## Abstract

**Background:**

Many women experience fear of childbirth (FoB) and depressive symptoms (DS) during pregnancy, but little is known about FoB among Tanzanian women. The current study aimed to assess the prevalence of FoB and DS among pregnant women and determine predictors of each and both, focusing on sociodemographic and obstetric predictors.

**Methods:**

A cross-sectional study was conducted at six health facilities in two districts in Tanzania between 2018 and 2019. In total, 694 pregnant women with gestational age between 32 and 40 weeks and expecting vaginal delivery were consecutively recruited and assessed for FoB and DS. We collected data through interviews using 6 and 4-points Likert Scale of the Wijma Delivery Expectancy Questionnaire Version A and Edinburgh Postnatal Depression Scale, respectively. Women who scored ≥66 and ≥ 10 were categorised as having FoB and DS, respectively. We performed multivariable logistic regression to investigate the predictors of FoB and DS.

**Results:**

The prevalence rates of FoB and DS among pregnant women were 15.1 and 17.7%, respectively. FoB and DS were more likely in women aged above 30 years [Adjusted Odds Ratio (AOR) 6.29, 95%CI 1.43–27.84] and in single mothers (AOR 2.57, 95%CI 1.14–5.78). Women with secondary education and above (AOR 0.22, 95%CI 0.05–0.99) and those who had given birth previously (AOR 0.27, 95% CI 0.09–0.87) were less likely to have FoB in combination with DS Women who had previous obstetric complications, and those who did not receive any social support from male partners in previous childbirth were more likely to have FoB and DS. FoB was strongly associated with DS (AOR 3.42, 95%CI 2.12–5.53). DS only was more common in women who had inadequate income (AOR 2.35, 95%CI 1.38–3.99) or had previously experienced a perineal tear (AOR 2.32, 95%CI 1.31–4.08).

**Conclusions:**

Not having a formal education, having only primary education, being aged above 30 years, being single, being nulliparous, having experienced obstetric complications, and having a lack of social support from a male partner during previous pregnancy and childbirth were predictors of FoB and DS during pregnancy. FoB and DS were strongly associated with each other. It is vital to identify at-risk women early, to offer support during pregnancy and childbirth.

**Supplementary Information:**

The online version contains supplementary material available at 10.1186/s12884-021-04169-7.

## Introduction

Pregnant women have predetermined childbirth expectations that could influence childbirth experience as they are approaching birth [1]. Childbirth experiences are multidimensional and unique to every woman, and can either be positive, negative, or a mixture of both. The experience can be influenced by individual factors such as: a woman’s own emotions and the freedom to express these emotions in relation to childbirth [2]; perceptions of, and personal involvement during birth process, preferences of the mode of delivery whether vaginal or caeserean section (C/S) [2–4]; and one’s own mental and physical health [5]. Other external related factors may involve and interaction with health care providers, support from a partner and/or relatives and lack of privacy during childbirth [2]. All of these features have a significant effect on the childbirth experience as some women can develop fear of having more children in the future [6–8].

Pregnancy as a transition period is accompanied by physical and emotional changes that can substantially affect the expectant mothers’ well-being [9]. Pregnancy related health problems can be due to previous existing heath conditions, whilst other problems arise during and shortly after pregnancy [10]. Common conditions such as anaemia, urinary tract infection, hypertensive disorders of pregnancy, gestational diabetes, anterpartum hemorrhage and hyperemesis gravidarum have been widely studied during and post pregnancy [11, 12]. Furthermore, a review from WHO on prevalence and determinants of common perinatal mental disorders stated that women from low- and lower-middle-income countries are at an increased risk of reporting psychological disorders during pregnancy and after childbirth [13]. The common perinatal mental disorders such as mood disorders, particulary perinatal depression and anxiety disoders, have been studied, but gaps in the literature exist in low-middle income countries, including Tanzania [14, 15]. Fear of childbirth (FoB) for the upcoming birth described under specific phobia in anxiety related disorders has been far less studied in pregnant women as compared to general anxiety and depression [16–18]. There is an association between FoB, depressive symptoms (DS) and other anxiety related problems, [19] and in some women they comorbid [20].

The prevalence of antenatal FoB varies between countries. Worldwide, FoB ranges between 5 and 30% [21–23] and DS affect 5–34% of women during pregnancy [24–26]. The rates in the Sub-Saharan African countries are reported to be 24.5% in Ethiopia [27], 22% Malawi [28] and 22.1% from Kenya [29]. However, the prevalence of DS is slightly lower among researched countries in Africa including rates from Malawi 19% [30], Ethiopia 19.9% [31], and Nigeria 24.5% [32]. In Tanzania, some studies have assessed DS during pregnancy, revealing high prevalence rates ranging from 11.5% in the Kilimanjaro region, northern Tanzania [33], to 33.8% in the Mwanza region [34]. Nevertheless, FoB and DS are not stable constructs, they vary depending on the pregnancy trimesters. In some women they may increase in late pregnancy/third trimester [35–37] or may be the same in early and late pregnancy [38, 39].

FoB and DS are due to various factors, which vary across countries. Quantitative studies across the world have shown that factors associated with FoB include sociodemographic factors such as young maternal age, lack of social support from male partners, relatives and friends, unemployment, financial constraints, and a history of abuse [40, 41]. Obstetric factors like nulliparity and adverse obstetric events, for example previous operative birth, may provoke childbirth fear [42–44]. Additionally, not being mentally and/or physically prepared for giving birth, an expectation of unendurable pain, a feeling of loss of control during labour and birth, and fear of death have been deemed to predict FoB [42, 43]. Inappropriate support from the birth team contributes to low childbirth confidence among women [43, 45].

In previous studies, DS were associated with unwanted pregnancy [46], young age/teenage pregnancy [37], low levels of education, unemployment, and low prestigious employment [46–48]. Inadequate social support (emotional, physical, and financial) and poor relationships with spouse/partner such as existing conflicts regarding sexual practices [34, 37, 46, 48], insufficient care, infidelity and polygamy, [34, 49] are other factors contributing towards development of DS among women.

FoB can be associated with pre-existing psychological problems like DS. An association between FoB and depression has been reported in a register study of 788,317 pregnant women in Finland, where depression was found to be the most potent risk factor for FoB among pregnant women, regardless of parity [50]. These results were similar to the findings from other studies performed around the world [51, 52].

Fear of childbirth may have consequences in the lives of women and their babies. It affects emotional and psychological well being of women [20, 40]. During childbirth, it can lead to increased use of pharmacologic pain relief [20], prolonged labour [53], aggravated maternal and foetal distress [20], and increased obstetric interventions, like labour argumentation [54] and C/S on maternal request [6, 55]. Maternal DS can interfere with women’s daily activities, mother-newborn bonding, early interruption of exclusive breastfeeding and/or discontinuation of breastfeeding and, for the infant, disruption of sleeping patterns and delays in cognitive development [56–58].

Tanzania is a multi-ethnic and multi-lingual country and has recently been upgraded into a middle-income country. There has been a significant socioeconomical transformation within its boarders following this economic scale up. This transformation may pose challenges to women of child bearing age, considering that financial difficulties are among the factors associated with FoB and other psychological problems. Moreover, the nature and scope of mental health issues among Tanzanian women may differ from women with western cultural backgrounds, norms perceptions and social structures and this would also affect classifications, diagnosis, and management of such illnesses. It is therefore essential to increase understanding of FoB and DS during pregnancy to enhance the chances of providing the right support to the mothers. To the best of our knowledge, studies in Tanzania on perinatal mental health are limited and no published studies have assessed FoB and its predictors among Tanzanian women, despite the advocacy on integrated mental health in maternal and child health services in the health system and proven long-lasting consequences of maternal FoB and/or DS. Studies on the prevalence of FoB and DS, their predictors, effects, and management, have been performed mainly in European countries, with a few from Asia [59] and Africa [27–29]. Hence, this study’s primary objective was to determine the prevalence of FoB and DS among pregnant women at ≥32 weeks of gestation. The secondary objective was to investigate the predictors of FoB and DS, focusing on sociodemographic factors and previous obstetric experiences.

## Methods

### Study design

This study was accomodated in a longitudinal study. Pregnant women were recruited and interviewed during the antenatal period and followed up through childbirth and interviewed again during the postnatal period. The present study investigated prevalence and predictors of FoB and DS among pregnant women in Pwani region, Tanzania.

### Study setting

We carried out the study in the Mkuranga and Kisarawe districts in the Pwani region, Tanzania. The country has seven geographical zones that are subdivided into 31 administrative regions. Pwani is one of the regions located in the Eastern zone with seven districts namely Mkuranga, Kisarawe, Bagamoyo, Kibaha, Mafia, Rufiji and Kibiti [60]. The Mkuranga district has 57 health facilities in total: 1 hospital, six health centres (2 governmental and four privately owned), 50 dispensaries (37 governmental and 13 privately owned). The Kisarawe district has a total of 40 health facilities, including 1 district hospital, 3 health centres, and 36 dispensaries (32 are governmental and four privately owned) [61]. In each district, one district hospital and two health centres, all of which were government-owned, were selected for the study. The selected health facilities had a volume of at least ten antenatal visits per day, and the district hospitals had facilities for childbirth by both vaginal delivery and C/S. In Tanzania, antenatal care is accessible in every public health care facility at no charge. A pregnant woman is recommended to make at least eight antenatal visits at these facilities prior to delivery; at these facilities where nurse-midwives are the primary care providers. In case of any complications identified when providing services, the nurse-midwife refers the woman to a specialist or higher-level health facility for further management.

### Study participants and procedure

We recruited and interviewed a consecutive sample of pregnant women seeking antenatal services at selected health facilities from September 2018 to March 2019. The eligibility criteria were: being pregnant at least the 32nd week of gestation; speaking Kiswahili; being resident in the selected district; having had no previous C/S; anticipating vaginal birth; attending the relevant health facility for antenatal care. The sample size was computed by using two proportion formula in Epi Info 7 StatCal with a power of 80% and a significant level of 0.05 with two tails. A minimum sample of 616 women was expected to be enrolled in this study. Of the 1130 pregnant women approached, 702 were invited to the study, eight refused participation, and 694 provided survey data. Women who refused to participate did so due to lack of time, having other commitments after receiving antenatal care or that their male partners were waiting for them; some women felt unwilling to take part in the survey.

We trained six registered nurse-midwives who were not employed during data collection as research assistants (RAs) in the data collection tools, use of a visual scale, study participant recruitment, and data collection procedures, including ethical principles in data collection. During the actual data collection, RAs recruited study participants, obtained informed consent, and performed data collection through face-to-face interviews in the selected antenatal clinics. We spent between 30 and 45 min per interview. Interviews were selected as the data collection method, to maintain data collection consistency, overcome the illiteracy challenge, and enhance comprehension of the questions and data collection scales. To decrease interview bias, participants could use a visual analogue scale to rate levels of fear for the Wijma Delivery and Expectation Questionnaire version A (W-DEQ-A). This reduced the risk of having different verbal explanations for response items from different research assistants. To enhance validity, all interviews were conducted in Kiswahili, and the quality of collected data was ensured by direct supervision of the first author and general guidance from all team members during the data collection.

### Instruments and measures

The participants were interviewed regarding sociodemographic (age, education level, occupation, income and marital status) and obstetric characteristics (gravidity, parity, pregnancy status, and complications on previous pregnancy). Further, we interviewed participants on expectations of childbirth, using the W-DEQ-A [62] and DS using the Edinburgh Postnatal Depression Scale (EPDS) [63]. Questions on obstetrics and social support were asked in relation to the history of the most recent previous pregnancy and childbirth.

The W-DEQ-A is a 6-point Likert scale questionnaire with 33 items ranging from 0 (not at all) to 5 (extremely), yielding a minimum score of 0 and a maximum score of 165. The higher the score, the more intense the FoB. The items refer to cognitive and emotional expectations of the upcoming childbirth (e.g. responding to the question, “*What do you think you will feel during labour and delivery”* with embedded words indicating opposite extremes of the expectations (e.g. “*Extreme panic”* vs. *“No panic at all” or “Extreme hopelessness”* vs. *“No hopelessness at all”, “Extreme self-confidence”* vs. *“No self-confidence at all”, “Extreme trust vs. No trust at all”, “Extreme pain vs. No pain at all”*). The cut-off points from the original tool areas follows: a score of ≤37 is considered a low level of fear, a score of 38–65 reflects a moderate level of fear, a score of 66–84 signifies a high level of fear, and a score of ≥85 indicates a severe level of fear [62, 64]. We defined high FoB as scoring ≥66 and low FoB as scoring < 66 [64, 65]. In this study no FoB will be referring to a low FoB and presence of FoB as a high FoB. The original W-DEQ-A has been reported to be a reliable questionnaire with a Cronbach’s alpha of 0.93. The original English questionnaire version was translated into Kiswahili and reviewed by a team of native Kiswahili speakers and professionals in midwifery, obstetrics, behavioral science, psychology, and psychiatry. Further, a pilot study and validation process were carried out and found that the translated W-DEQ-A was a reliable tool, with a Cronbach’s alpha of 0.83 (Massae, Larsson, Leshabari, Mbekenga, Pembe and Svanberg. Fear of childbirth: Validation of the Kiswahili version of Wijma delivery expectancy/experience questionnaire version A&B in Tanzania. Forthcoming).

The EPDS is a tool used to identify common perinatal mental disorders and has been used to screen women at risk of developing depression during pregnancy or/and after childbirth [63]. The EPDS is a four-point Likert scale with 10 items ranging from 0 to 3. Participants rate each item based on how they have felt in the preceding seven days with items like: *I have been able to laugh and see the funny side of things* with responses “As much as I always could,” “Not quite so much now”, “Definitely not so much now”, “Not at all”. Other items were *I have looked forward with enjoyment to things, I have blamed myself unnecessarily when things went wrong, I have been anxious or worried for no good reason, I have felt scared or panicky for no very good reason, Things have been getting on top of me, I have been so unhappy that I have had difficulty sleeping, I have felt sad or miserable, I have been so unhappy that I have been crying, The thought of harming myself has occurred to me*. The lowest score that can be obtained on this scale is 0, while the highest is 30. The higher the score, the higher the risk of having DS. Scores ≥10 suggest the presence of DS, while scores ≥13 represent depressive illness of varying severity [63]. In this study, we defined DS as scoring ≥10 and no DS as scoring < 10 [17, 30, 66]. The internal consistency reliability of the original EPDS was excellent, with a Cronbach’s alpha of 0.88. Our study had a Cronbach’s alpha of 0.85. The EPDS has been translated into Kiswahili in Kenya and showed an acceptable Cronbach’s alpha of 0.78 [25]. We adapted the EPDS from Kenya, translated by Green and colleagues [25], for data collection in Tanzania.

### Data analysis

Data were analysed using the Statistical Package for the Social Sciences (SPSS) version 26. Descriptive statistics were used to summarise demographics, obstetric characteristics, and the prevalence rates of FoB and DS. A chi-squared test was performed to determine the associations between sociodemographic and obstetric variables and the prevalence rates of FoB and DS. Logistic regression was used to estimate the effects of sociodemographic and obstetric variables on the prevalence rates of FoB and DS after adjusting for potential confounders. Univariable analysis was conducted to assess the association between each sociodemographic and obstetric variable and the presence of FoB and/or DS. All the variables with *p* < 0.2 in the univariable analysis were subjected to several multivariable logistic regression models using an ENTER approach where variables were entered into the models, one after another. Age, education, and parity were included in every model, since they had been shown to be confounders of FoB and DS predictors in previous studies. A *p*-value of < 0.05 was considered significant. Effect sizes were presented using odds ratios (ORs) with 95% confidence intervals (CIs). Multicolliniarity of independent variables was assessed using Variance Inflation Factor (VIF). Variables with VIF ≤ 5 were maintained for further analysis. The outcomes of interest were grouped as (1) FoB vs.no FoB and DS vs. no DS, (2) Neither FoB nor DS vs. FoB but no DS, DS but no FoB, and FoB in combination with DS.

## Results

### Description of the population

In total, 694 eligible pregnant women agreed to participate in this study. The response rate was 98.9%. Their age varied between 14 and 46 years, with a median age of 26 years and IQR of 11 and the majority (73.2%) were married and had primary education (61.8%). The majority were financially (59.1%) and socially (36.9%) supported by their male partners and had planned the pregnancy.

### Prevalence of FoB and DS

The total scores for FoB ranged from 1 to 102, while those for DS were 0–27. The median scores with interquartile range (IQR) for FoB and DS were 48 (24.0) and 3 (7), respectively. The overall prevalence of FoB was 15.1% and that of DS was 17.7%. The results indicated that about 37 (5.3%) of the pregnant women had both FoB and DS, 68 (9.8%) had FoB but no DS, 86 (12.4%) had DS but no FoB, and 503 (72.5%) had neither FoB nor DS.

#### Scores forthe item in the W-DEQ-A

Scores for each item in the W-DEQ-A are shown in Table 1 in descending order of prevalence. The most common fear reported by pregnant women was labour pain. The items for which most women scored low were longing for a child and childbirth being fun.

**Table 1:** Fear of childbirth scores by item

**Item number**	**Items**	**Mean (Standard deviation)**	**Total scores per item**
24	Painful	4.2 (0.9)	2918
25	Behave badly	3.1 (1.6)	2147
2	Frightful	2.8 (1.6)	1955
3	Lonely	2.8 (1.6)	1913
6	Afraid	2.7 (1.6)	1883
19	Panic	2.7 (1.5)	1884
26	Surrender control of the body	2.4 (1.7)	1651
12	Tense	2.5 (1.5)	1768
8	Weak	2.3 (1.5)	1616
31	Dangerous	2.1 (1.6)	1446
27	Lose control	1.8 (1.4)	1269
7	Deserted	1.7 (1.4)	1213
15	Abandoned	1.7 (1.5)	1198
11	Desolate	1.6 (1.4)	1114
1	Fantastic	1.5 (1.5)	1019
13	Glad	1.5 (1.4)	1077
20	Hopelessness	1.5 (1.6)	1066
18	Happy	0.9 (1.2)	603
17	Relaxed	0.8 (1.1)	549
32	Fantasy thatchild will die	0.8 (1.1)	551
4	Strong	0.7 (0.9)	478
9	Safe	0.7 (1.0)	480
10	Independent	0.7 (1.1)	507
14	Proud	0.7 (1.1)	458
16	Composed	0.7 (1.0)	506
33	Fantasy that child will be injured	0.7 (0.9)	453
5	Confident	0.6 (0.9)	399
29	Natural	0.5 (0.9)	365
30	Self-evident	0.5 (1.0)	344
22	Self confidence	0.4 (0.8)	289
23	Trust	0.4 (0.8)	274
21	Longing for a child	0.1 (0.5)	55
28	Funny	0.1 (0.5)	73
**Total fear of childbirth score**		**48.2 (16.3)**	**33,521**

### Association between demographic and obstetric variables and FoB and/or DS

Education level, marital status, sex of the baby, male partner social support, and experience of obstetric complications were significant variables associated with FoB (*p* < 0.05). DS and FoB were strongly associated (*p* < 0.001). DS was associated with education level, marital status, income adequacy, previous perineal tear, and previous obstetric complication (Table 2).

**Table 2:** Associations of sociodemographic and obstetric variables with fear of childbirth and depressive symptoms

**Variables**	**Total**	**Fear of childbirth (W-DEQ-A)**			**Depressive symptoms (EPDS)**		
		**No fear (≤65)**	**Fear (≥ 66)**	***P*-value**	**No Depessive symptoms (< 10)**	**Depressive symptoms (≥ 10)**	***P*value**
	**n (%)**	**n (%)**	**n (%)**		**n (%)**	**n (%)**	
**Index pregnancy variables (*n*** **= 694)**							
**Age group**				0.371			0.235
≤ 20 years	139 (100)	113 (81.3)	26 (18.7)		118 (84.9)	21 (15.1)	
21–30 years	358 (100)	305 (85.2)	53 (14.8)		286 (79.9)	72 (20.1)	
≥ 31 years	197 (100)	171 (86.8)	26 (13.2)		167 (84.8)	30 (15.2)	
**Education level**				0.121			0.056
No formal education	137 (100)	116 (84.7)	21 (15.3)		116 (84.7)	21 (15.3)	
Primary education	429 (100)	357 (83.2)	72 (16.8)		359 (83.7)	70 (16.3)	
Secondary and higher	128 (100)	116 (90.6)	12 (9.4)		96 (75.0)	32 (25.0)	
**Occupation**				0.446			0.687
Employed	490 (100)	419 (85.5)	71 (14.5)		405 (82.7)	85 (17.3)	
Not employed	204 (100)	170 (83.3)	34 (16.7)		166 (81.4)	38 (18.6)	
**Marital status**				**0.048**			**0.003**
Married	581(100)	500 (86.1)	81 (13.9)		489 (84.2)	92 (15.8)	
Single	113 (100)	89 (78.8)	24 (21.2)		82 (72.6)	31 (27.4)	
**Income**				0.286			**0.001**
Adequate	304 (100)	263 (86.5)	41 (13.5)		267 (87.8)	37 (12.2)	
Inadequate	390 (100)	326 (83.6)	64 (16.4)		304 (77.9)	86 (22.1)	
**Gravidity**				0.244			0.551
Primigravida	161 (100)	132 (82.0)	29 (18.0)		135 (23.6)	26 (21.1)	
Multigravida	533 (100)	457 (85.7)	76 (14.3)		436 (76.4)	97 (78.9)	
**Parity**				0.244			0.196
Nulliparous	347 (100)	289 (83.3)	58 (16.7)		279 (80.4)	68 (19.6)	
Parous	347 (100)	300 (86.5)	47 (13.5)		292 (84.1)	55 (15.9)	
**Pregnancy status**				0.693			0.165
Planned	471 (100)	398 (84.5)	73 (15.5)		381 (80.9)	90 (19.1)	
Unplanned	223 (100)	191 (85.7)	32 (14.3)		190 (85.2)	33 (14.8)	
**DS during pregnancy**				**< 0.001**			
No	571 (100)	503 (88.1)	68 (11.9)		NA		
Yes	123 (100)	86 (69.9)	37 (30.1)		NA		
**FoB during pregnancy**							**< 0.001**
No	589 (100)	NA			503 (85.4)	86 (14.6)	
Yes	105 (100)	NA			68 (64.8)	37 (35.2)	
**Previous birth and pregnancy variables (*n*** **= 504)**							
**Social support from a male partner in previous childbirth**				**0.043**			0.311
Yes	376 (100)	328 (87.2)	48 (12.8)		309 (82.2)	67 (17.8)	
No	128 (100)	103 (80.5)	25 (19.5)		100 (78.1)	28 (21.9)	
**Previous perineal tear**				0.228			**0.006**
Yes	130 (100)	107 (82.3)	23 (17.7)		95 (73.1)	35 (26.9)	
No	374 (100)	324 (86.6)	50 (13.4)		314 (84.0)	60 (16.0)	
**Ever experienced obstetric complications**				**0.007**			**< 0.001**
Yes	141 (100)	111 (78.7)	30 (21.3)		104 (73.8)	37 (26.2)	
No	363 (100)	320 (88.2)	43 (11.8)		305 (84.0)	53(16.0)	

### Regression analysis of the predictors of FoB and DS

Tables 3 and 4 show different logistic regression models for both univariable and multivariable analyses of FoB and DS as outcomes. Experience of obstetric complications in a previous pregnancy and birth was the strongest predictor of FoB. Another strong predictor of FoB was having a low educational level and lack of social support from a male partner in a previous childbirth. The strongest predictors of DS were being single, inadequate income, lack of social support from a male partner in a previous childbirth, having ever experienced obstetric complications, and have experienced a perineal tear before the index pregnancy (Table 3).

**Table 3:** Demographic and obstetrics predictors of fear of childbirth and depressive symptoms

**Variables**	**Fear of childbirth**		**Depressive symptoms**	
	***n* = 105/694 (15.1%)**		***n* = 123/694 (17.7%)**	
	**Unadjusted**	**Adjusted**	**Unadjusted**	**Adjusted**
	**OR (95% CI)**	**OR (95% CI)**	**OR (95% CI)**	**OR (95% CI)**
**Age**				
≤ 20 years	1	1	1	1
21–30 years	0.76 (0.45–1.27)	1.02 (0.55–1.89)	1.32 (0.76–2.29)	1.38 (0.70–2.69)
≥ 31 years	0.66 (0.37–1.19)	0.88 (0.38–2.03)	0.93 (0.49–1.73)	1.05 (0.44–2.53)
**Education level**				
No formal education	1	1	1	1
Primary education	1.11 (0.66–1.89)	0.94 (0.54–1.64)	1.08 (0.63–1.83)	0.90 (0.51–1.61)
Secondary education and above	0.58 (0.27–0.99)*	0.42 (0.18–0.94)*	1.84 (0.99–3.40)	1.49 (0.72–3.09)
**Parity**				
Nulliparous	1	1	1	1
Parous	0.78 (0.51–1.19)	0.78 (0.42–1.45)	0.77 (0.52–1.14)	0.79 (0.43–1.44)
**Marital status**				
Married	1	1	1	1
Single	2.01 (1.33–3.02)*	1.56 (0.92–2.65)	2.01 (1.33–3.02)*	1.72 (1.01–2.91)*
**Planned pregnancy**				
No	1		1	
Yes	1.09 (0.69–1.72)	NA	1.36 (0.88–2.10)	NA
**Income**				
Adequate			1	1
Inadequate	1.26 (0.82–1.93)	NA	2.04 (1.34–3.10)*	2.39 (1.49–3.83)**
**Social support from a male partner in a previous childbirth**				
No	1	1	1	1
Yes	0.75 (0.49–1.14)	0.49 (0.29–0.79)*	0.71 (0.48–1.04)	0.61 (0.37–0.98)*
**Ever experienced obstetric complications**				
No	1	1	1	1
Yes	1.72 (1.72–2.61)*	2.03 (1.24–3.23)*	2.33 (1.43–3.81)*	1.76 (1.07–2.89)*
**Previous perineal tear**				
No	1		1	1
Yes	1.26 (0.76–2.09)	NA	2.03 (1.26–3.25)*	1.21(0.72–2.01)
**DS**				
No	1		**NA**	NA
Yes	3.18 (2.01–5.05)**	3.42 (2.12–5.53)**	**NA**	NA
**FoB**				
No	NA	NA	**1**	**1**
Yes	NA	NA	3.18 (2.01–5.05)**	3.23 (2.01–5.35)**

*NA* Not applicable to that particular category and not entered into the multivariable model as the univariable pvalue was ≥0.200

*p < 0.005, **p < 0.001

**Table 4:** Predictors of having DS or FoB only or both

**Variables**	**Predictors of having fear of childbirth only**		**Predictors of having depressive symptoms only**		**Predictors of having both fear of childbirth and depressive symptoms**	
	***n* = 68/571 (11%)**		***n* = 86/589 (14.6%)**		***n* = 37/540 (6.9%)**	
	**Unadjusted**	**Adjusted**	**Unadjusted**	**Adjusted**	**Unadjusted**	**Adjusted**
	**OR (95%Cl)**	**OR (95%Cl)**	**OR (95%Cl)**	**OR (95%Cl)**	**OR (95%Cl)**	**OR (95%Cl)**
**Age**						
≤ 20 years	1	1	1	1	1	1
21–30 years	0.78 (0.42–1.43)	0.69 (0.33–1.42)	1.66 (0.87–3.17)	0.89 (0.41–1.94)	0.89 (0.38–2.13)	2.56 (0.95–6.89)
≥ 31 years	0.55 (0.26–1.14)	0.39 (0.15–1.04)	1.96 (0.46–2.03)	0.59 (0.22–1.62)	0.91 (0.35–2.33)	6.29 (1.43–27.84)*
**Education level**						
No formal education	1	1	1	1	1	1
Primary education	1.04 (0.55–1.98)	1.32 (0.51–3.45)	0.99 (0.52–1.89)	0.36 (0.16–0.81)*	1.25 (0.53–2.96)	0.95 (0.37–2.43)
Secondary and higher	0.75 (0.31–1.83)	1.33 (0.61–2.94)	2.43 (1.21–4.87)*	0.34 (0.19–0.63)*	0.50 (0.13–2.00)	0.22 (0.05–0.99)*
**Parity**						
Nullipara	1	1	1	1	1	1
Multipara	0.84 (0.57–1.58)	1.42 (0.69–2.88)	0.91(0.57–1.43)	1.33 (0.68–2.60)	0.51 (0.26–1.03)	0.27 (0.09–0.87)*
**Income**						
Adequate	1	1	1	1	1	1
Inadequate	1.13 (0.68–1.88)	NA	2.06 (1.26–3.36)*	2.35 (1.38–3.99)*	2.11 (1.02–4.35)*	2.09 (0.97–4.52)
**Marital status**						
Married	1	1	1	1	1	1
Single	1.33 (0.68–2.59)	NA	2.04 (1.27–3.29)*	1.98 (1.18–3.32)*	**2.97 (1.43–6.18)**	2.57 (1.14–5.78)*
**Ever experienced obstetric complications**						
No	1	1	1	1	1	1
Yes	1.15 (0.69–1.91)	NA	1.32 (0.84–2.09)	NA	4.20 (1.88–9.37)*	3.19(1.42–7.19)*
**Planned pregnancy**						
No	1	1	1	1	1	1
Yes	1.23 (0.71–2.13)	NA	1.58 (0.93–2.67)	NA	1.06 (0.52–2.17)	NA
**Previous perineal tear**						
No	1	1	1		1	1
Yes	1.49 (0.80–2.78)	NA	2.42 (1.45–4.05)*	2.32 (1.31–4.08)*	1.46 (0.64–3.31)	NA
**Social support from a male partner**						
No	1	1	1	1	1	1
Yes	1.52 (0.91–2.52)	NA	1.59 (1.00–2.51)*	1.46 (0.87–2.45)	1.29 (0.66–2.52)	NA

*NA* Not applicable – indicates that the univariable *p* value was ≥0.20 and that the variable was not entered into the multivariable model

*p < 0.005, **p < 0.001

As shown in Table 4, the predictors of DS only, as compared with no FoB or DS were: lacking formal education; being unmarried; having inadequate income; having experienced a perineal tear in a previous delivery. The odds of having both FoB and DS as compared with no FoB and no DS were higher in older, single, and/or nulliparous women, those with a low education level, and those who had experienced obstetric complications.

See Additional file 1.

## Discussion

We aimed to determine the prevalence rates and predictors of FoB and DS among pregnant women attending antenatal clinics at selected public health facilities. We found that the prevalence rates of FoB and DS were in a similar range: 15.1 and 17.7%, respectively. Some women had both FoB and DS, some had FoB but no DS or DS but no FoB, and others had neither FoB nor DS. Predictors of FoB and DS were lack of formal education, age above 30 years, never having given birth, being single, having had obstetric complications in the past, and having experienced a lack of male social support at a previous childbirth. Predictors of FoB were not having a formal education, while DS was seen in women without formal education, with inadequate income, who were single, and/or who had experienced a tear/episiotomy in a previous childbirth. Another key finding was that FoB and DS were strongly associated.

The prevalence rates of FoB and DS among Tanzania pregnant women in our study are within global ranges when the same validated tools are used for analysis [24, 25, 28, 33, 34, 59, 67]. The similar results across the globe might indicate that most women fear labour pain, being alone, and losing control of their body during labour and childbirth. Tanzanian women, like other women worldwide, have positive expectations on health facility childbirth [68]. Most women who prefer health facility childbirth seek life-saving technology in case of obstetric complications. Some wish to have family members nearby to provide care and affection [69]. However, Tanzanian women’s expectations may be hard to meet due to the high patient-health care provider ratio at most public health facilities [70]. This could contribute to the high prevalence rates of FoB and DS. Also, disrespectful and abusive treatment from health providers during childbirth has been reported in Tanzania. For instance, women report being neglected/ignored, receiving physical and verbal abuse [71], being shouted at, threatened, slapped/pinched, or being left alone and forced to deliver by themselves [72]. All of these events could cause FoB and DS.

Being above 30 years of age, not having a formal education or having primary education only, being single, being nulliparous, and having previous experience of obstetric complications were common among women with FoB in combination with DS. We found that older women were more fearful and had more DS than younger ones. The findings were in line with those of a study done in Norway, assessing childbirth experiences in first-time mothers of advanced age [73]. The reason could be that older women have heard of more complications, which might intensify fear ahead of childbirth. Another reason could be, older women have been exposed more to the childbirth process, and some might have gone through negative childbirth experience.

We found contradictory findings, showing that younger women/teenagers were more likely to develop DS and FoB than older women [41]. The discrepancy could be due to most teenagers being nulliparous with limited childbirth experience, which could manifest as DS and FoB. Health care providers should be attentive to all women and their differing childbirth expectations. FoB and DS were more likely in single women than married women. These findings were similar to those of previous studies [29, 32, 46].

Further, women who had never given birth previously had a higher risk of having both FoB and DS. This was in line with other studies, which reported that nulliparous women presented with FoB more often than multiparous women [6, 44]. However, we could not find any study assessing predictors of FoB in combination with DS for comparison therefore further studies are needed.

Previous obstetric complications were strong predictors of both FoB and DS. Similar findings have been made in several other studies: a previous negative experience predicts FoB and DS in subsequent childbirth [6, 74, 75]. Negative birth experiences can lead to hesitance at becoming pregnant or giving birth in the future, resulting in delaying subsequent pregnancies or total avoidance of later pregnancies [22, 76]. This might impact on women’s future reproductive lives, likely diminishing trust in the ability to give birth and trust in maternity services. Such mistrust might lead to women not seeking maternity services from health facilities or opting for a C/S rather than vaginal birth [77, 78]. It is crucial to raise nurse-midwives awareness on the possible causes of negative birth experiences and to discuss how to support these women during subsequent pregnancies and childbirths. Women who have negative experiences should be identified during antenatal care for psychological support during pregnancy and childbirth. The same applies for women at risk of experiencing obstetric complications. These women will need to be empowered through provision of antenatal education on what to expect throughout the perinatal period.

In this study, women who received social support from their male partners during previous childbirths were less likely to report FoB and DS. This is in line with previous studies which reported that male partner support during childbirth is essential in alleviating fear related to childbirth [41] and preventing DS [79]. This highlights the importance of partner companionhip before, during, and after childbirth in a country where a male companion is not yet allowed or standard procedure during health facility childbirth.

Our study showed that FoB and DS were strongly associated. DS during pregnancy predicted having FoB. These findings are in line with findings from most systematic reviews and published studies [76, 80].

Depressive disorders are an important health problem globally [81, 82]. Among the participating women, DS was more likely to occur in women without formal education, who were single, who had an inadequate income and/or who had experienced tearing and/or an episiotomy in aprevious delivery. Our findings were in line with previous studies showing that single mothers [46, 83] and those with inadequate income [34, 46] had increased risks of DS. Further, several previous studies have found that less educated people are more likely to be depressed than more educated ones [46, 84]. There might be a pattern in how people of a similar educational background perceive the childbirth process, child upbringing, and associated resources. Another explanation could be that the more educated people are, the more they seek information from different sources, which could prevent them from emotional distress, manifesting as DS. Our findings were also consistent with another study showing that perineal wounds due to either tearing or episiotomy were associated with DS [59].

### Strengths and limitations

The study’s major strength was the use of standardised, validated, and widely used tools for measuring FoB (W-DEQ-A) and DS (EPDS), increasing credibility. The use of a visual aid scale was an added advantage that ensured clarity when using the W-DEQ Likert scale in the W-DEQ tool. Further, the sample was large enough to determine the prevalence rates and predictors of the outcome variables. While many other similar studies have used self-administered tools, we used the interviewer-administered questionnaire technique, eliminating the hurdle for participants strugglingto fill in responseson their own.

Using interviewers for data collection was a strength, but could also entail some limitations. If women adjusted their responses to please the interviewer, a social desirability bias might have occurred. This could lead to the prevalence rates of FoB and DS being slightly under estimated. Being a cross-sectional study could be a limitation in finding predictors since the variables were assessed only at one single time. Another limitation was that we did not know whether women in our study who were categorised as having FoB and DS had received any treatment. We were also unable to offer any support to women identified as having any problems. This is because the collected data were analysed and interpreted weeks later, meaning that women could not be referred for further management. Furthermore, the tools were only for screening purposes and therefore could not be used for clinical diagnosis. Our findings cannot be generalised to the first and second trimester of pregnancy, as data were collected duringthe third trimester. Comparisons with results from other studies may be impaired because of different data collection tools and cut-off points used to measure FoB and DS. However, we compared our findings with other studies that used only W-DEQ and EPDS tools to define FoB and DS [64] and DS [63], leaving out studies that used other tools to measure FoB and DS. W-DEQ-A might have been affected by being translated into Kiswahili, due to cultural diversity and differences in how women define and perceive childbirth in different countries. Furthermore, use of an adapted version of the EPDS validated in Kenya, where Kiswahili is also a national language, might be a limitation, as some words may have a different meaning when used in Tanzania.

## Conclusions

Previous obstetric complications were the strongest predictor of FoB and DS, and FoB and DS were strongly associated with each other. Lack of social support from a male partner was also a predictor of FoB and DS. Not having a formal education was a predictor of FoB. Being single, having an inadequate income and having experienced a perineal tear were strong predictors of DS. Having FoB in combination with DS was more commonin women aged above 30 years, without formal education, who were nulliparous and/or single. Understanding why some women are more prone to FoB and DS is vital in developing effective prevention and timely intervention to restore their mental health and psychological well-being throughout pregnancy, delivery, and after childbirth. This could, in turn, decrease mental suffering and negative consequences linked to FoB and DS.

Clinical implications from our results could be the possibilities for: a) proper screening of pregnant women for FoB and DS during the antenatal period; b) identifying and providing support to women at risk of developing mental illnesses and those who have experienced complications during previous pregnancies and childbirths.

## Supplementary Information

**Additional file 1.**

## Data Availability

The datasets used and/or analysed during the current study are available from the Director of research and publication of Muhimbili Univeristy and Allied Sciences.

## Abbreviations

AOR: Adjusted odds ratio
CI: Confidence interval
DS: Depressive symptoms
EPDS: Edinburgh Postpartum Depression Scale
FoB: Fear of childbirth
IQR: Interquartile range
RAs: Research assistants
W-DEQ-A: Wijma Delivery Expectancy/Experience Questionnaire-A
