# Supplementary material for: Predictors of fear of childbirth and depressive symptoms among pregnant women: a cross-sectional survey in Pwani region, Tanzania
Source: BMC Pregnancy Childbirth. 2021 Oct 19;21:704. doi: 10.1186/s12884-021-04169-7 (PMC8524824; doi:10.1186/s12884-021-04169-7)
Supplement: Supplementary file 1 — Additional file 1. [file 12884_2021_4169_MOESM1_ESM.docx]

**Additional Information**

| **What is already known**  Nulliparous women report a higher level of fear of childbirth (FoB) than parous women. The Wijma Delivery Experience/Expectancy Questionnaire version A and Edinburgh Postnatal Depression Scale are the most frequently used tools for measuring FoB and DS, respectively, during pregnancy and have been validated worldwide.  **What this study adds**   1. This is the first study to investigate the prevalence and predictors of FoB in Tanzania. 2. Previous obstetric complications are the strongest predictor of both FoB and DS. 3. Lack of formal education, being aged above 30 years, being single, and being nulliparous are predictors of FoB in combination with DS. |
| --- |
